# Case Report: Capacity to Objectively Monitor the Response of a Chronic Pain Patient to Treatment

**DOI:** 10.3389/fnimg.2022.831216

**Published:** 2022-05-09

**Authors:** Julia Watson, Darren Lukas, E. Russell Vickers, Graham Galloway, Carolyn E. Mountford

**Affiliations:** ^1^Faculty of Health, School of Clinical Sciences, Queensland University of Technology, Brisbane, QLD, Australia; ^2^Princess Alexandra Hospital, Department of Radiology, Woolloongabba, QLD, Australia; ^3^Department of Imaging Technology, Translational Research Institute, Woolloongabba, QLD, Australia; ^4^Institute for Glycomics, Gold Coast Campus, Griffith University, Southport, QLD, Australia; ^5^Clinical Stem Cells Pty Ltd (PL), Sydney, NSW, Australia

**Keywords:** pain, response to therapy, neurochemistry, fucosylated glycan imbalance, glutathione redox imbalance

## Abstract

Response to pain therapy is currently by patient self-report. We demonstrate that by evaluating the neurochemistry of a patient, using two-dimensional Correlated SpectroscopY (2D COSY) in a 3T MRI scanner, response to therapy can be recorded. A chronic temporomandibular joint (TMJ) pain patient was evaluated by a pain physician specializing in temporomandibular disorders (TMD), and by 2D COSY, before, and 6 days after treatment with Botulinum Toxin A. Prior to treatment the self-reported pain score was 8/10 and reduced to 0/10 within 24 h of treatment. The neurochemistry of the patient prior to treatment was typical of chronic pain. In particular, the Fuc-α(1–2) glycans were affected. Following treatment, the substrates, α-L Fucose, were elevated and the Fuc-α(1–2) glycans repopulated. The depletion of the molecule assigned the glutathione cysteine moiety, with chronic pain, is indicative of a Glutathione redox imbalance linked to neurodegeneration. This new approach to monitor pain could help discriminate the relative contributions in the complex interplay of the sensory and affective (emotional suffering) components of pain leading to appropriate individualized pharmaceutical drug regimens.

## Introduction

The current “gold-standard” for pain assessment are self-report medical questionnaires. Necessarily, the response to the questionnaires are based on the patients' own perception of their pain and their ability to understand information and then communicate this individual experience (Cowen et al., 2015). Pain is an invisible phenomenon and if the person is unable to communicate, for example young children and infants, other physiological or behavioral signs are followed. In a similar manner, diagnosis of animals experiencing pain is by observational measures. The pain community recognizes the urgent need for objective markers of pain.

There are multiple studies using *in-vivo* neuro spectroscopy reporting alterations to neurochemistry as a consequence of chronic pain (Siddall et al., 2006; Stanwell et al., 2010). Changes recorded in participants with chronic pain, using 1D and 2D magnetic resonance spectroscopy, include the spectral regions consistent with the recently assigned substrate α-L Fucose and the Fuc-α(1–2) glycans in the human brain (Murrey et al., 2009; Mountford et al., 2015; Tosh et al., 2019). Currently there are seven fucose-α(1–2)-galactose sugars (glycans) and free α-L-fucose substrates that have been assigned using the 2D-L-COSY pulse sequence *in vivo* in the human brain (Tosh et al., 2019).

The Fuc-α(1–2) glycans have been shown in animal models, to be implicated in the mechanisms underlying neuronal development, learning, memory (Murrey et al., 2009); regulation of nervous system development (Murrey and Hsieh-Wilson, 2008); and to influence various neuronal processes (Ralf and Melitta, 2004; Murrey et al., 2009). Prior reports to evaluate pain associated with spinal cord injury (Stanwell et al., 2010) include this spectral region as a distinguishing feature. The ability to non-invasively study the fucose-α(1–2)-glycan residues in the brain is the key to being able to understand their function and deregulation.

Chronic temporomandibular joint (TMJ) disorders affect the masticatory muscles and temporomandibular joints causing pain and dysfunction (Rinchuse and Greene, 2018). Over 90% of people are affected by temporomandibular disorder including TMJ pain at some stage of their life, with a higher prevalence in females aged 20–40 (Laplanche et al., 2012). To ameliorate the pain one treatment is the injection of a therapeutic dose of botulinum toxin A, into the typically afflicted masticatory temporalis and masseter muscles. This can relieve jaw muscle spasm and relieve headaches due to bruxism (jaw clenching and teeth grinding).

Here we evaluate the neurochemistry of a patient suffering from chronic TMJ before and after treatment with botulinum toxin A using *in vivo* 2D correlated spectroscopy (COSY) in a clinical 3T MR scanner.

## Materials and Methods

### Ethical Approval

Institutional ethics approval was received from Queensland Health Metro South Ethics Committee (HREC/17/QPAH/808). All components of this study were conducted in accord with approved guidelines and regulations from relevant governance and institutional bodies. All participants in the study provided written informed consent.

### Patient

A 37-year-old woman with a six-year history of chronic TMJ was recruited for this study and examined by a clinician specializing in TMJ pain. The participant's data was statistically compared to *n* = 14 healthy controls (100% F, mean 35.71 ± S.D. 6.58). The patient had experienced a level of pain that made it difficult to concentrate and had a history of successful outcomes from treatment with Botulinum Toxin A, with a cessation of pain within 24 h.

### Healthy Control Cohort

The healthy controls were recruited from a number of sources including social media and local advertising. Participants were included if they were aged between 18 and 65 years. They were assessed by the Structured Clinical Interview for DSM V (SCID) 28 and included if they had had no current DSM-V Axis I disorder and no history of an anxiety or mood disorder, current or past history of neurological disease; major head injury; currently or possibly pregnant or had any contraindications to MRI scanning.

### Self-Report Measures and Clinical Interviews

Healthy controls were screened by a Clinical Psychologist using a combination of clinical interview and psychometric tests. These tests included a screen to assess symptoms of post-traumatic stress disorder (post-traumatic stress disorder checklist), mood disorders, anxiety disorders (depression, anxiety and stress scale), alcohol-related disorders (alcohol use disorders identification test) and history of head injury (Ohio State University TBI checklist). Following the psychological assessment, neuroimaging was conducted.

### MR Imaging and Spectroscopy

The TMJ participant was imaged prior to Botulinum Toxin A treatment and again 6 days after treatment. All scans were performed on a 3T Prisma scanner (Siemens, Erlangen, Germany, software version VD13D and VE11C) with a 64-channel head and neck coil (Siemens, Erlangen) at the Princess Alexandra Hospital (QLD, Australia).

### Structural Imaging

Magnetic resonance imaging including T1 and T2 weighted images were obtained for diagnostic reporting and anatomical localization. A 3D T1-weighted magnetization-prepared rapid gradient-echo (MPRAGE) was acquired (TR/TE/TI = 2,530/3.5/1,100 ms, flip angle = 7°, field of view = 256 × 256 mm, IPAT = 3, acquisition time 4:28 min), for accurate MRS voxel placement.

On T1 and T2 weighted imaging an 11 cm sebaceous cyst on the left side of the skull vault was identified. This was considered an incidental finding that was present on T1 weighted imaging at each time point.

### Localized COSY 2D MR Spectroscopy

**Figure 1:**
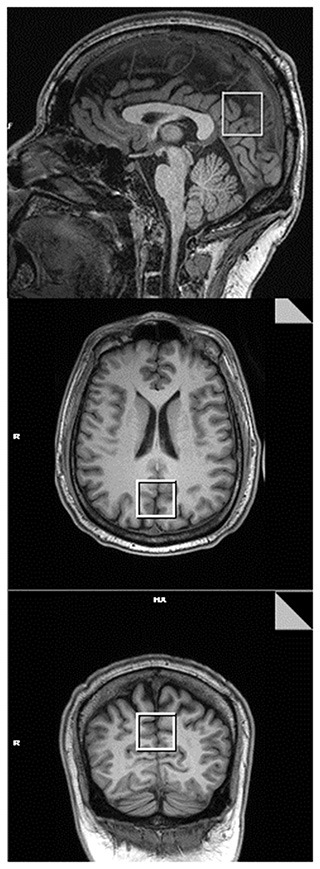
Representative T1 MPRAGE images taken in the mid-sagittal (Top), axial (Middle), and coronal (Bottom) planes. The box represents the voxel placement in the posterior cingulate gyrus (PCG) of the brain when using 2D COSY.

L-COSY data were acquired from a 3 × 3 × 3 cm^3^ voxel positioned in the posterior cingulate gyrus (PCG), Figure 1. In this article, as in previous work, data is collected from the PCG because it can be routinely shimmed to achieve sub-15 Hz linewidths from a large voxel. L-COSY was acquired with the following parameters: RF carrier frequency at 2.0 ppm; TR 1,500 ms; TE 30 ms; water suppression using WET; 96 t1 increments; with 8 averages per increment, acquired vector size 1,024 points; acquisition time 512 ms; spectral width in F2 2,000 Hz and spectral width in F1 1,250 Hz (0.8 ms increment size). Time of acquisition was 19 min, 12 s. Localized shimming was undertaken by adjustment of zero- and first-order shim gradients using the automatic B_0_ field mapping technique supplied by the vendor (Siemens AG). Each voxel was manually shimmed to ensure that FWHM was not >15 Hz as per the system reported results. All participant data obtained was acceptable for analysis.

### Evaluation and Statistical Analysis of the 2D L-COSY Data

Data was processed and analyzed using MATLAB (R2017b, The MathWorks, Inc., Natick, Massachusetts, USA), and FelixNMR (2007, San Diego, USA), a 2D NMR processing software. The post-processing parameters used in Felix were: F2domain (skewed sine-squared window, zero-filling to 2,048 points), F1 domain (sine-squared window, linear prediction to 96 points, zero-filling to 512 points) (Lin et al., 2021). In Felix, each prominent diagonal and cross peak was selected and integrated to determine the peak chemical shift, intensity, and volume. These values were internally normalized using the total creatine methyl diagonal peak at 3.02 ppm. Peak and cross-peak assignments were manually adjusted, to ensure the region of integration was centered on the peak, then exported for further analysis. Each spectrum was referenced to the creatine cross peak (3.02, 3.02 ppm) and specifying a constant number of contour levels (set to 28), as well as a constant level multiplier, set to 1.05 (Lin et al., 2021).

The average chemical shift, in ppm, of each cross-peak position from the 14 healthy volunteers was then calculated and compared to the patient before and after treatment. The *z*-score was calculated on the healthy cohort distribution (mean and standard deviation) to see how much the pre- and post-deviated from the healthy cohort. We took the peaks in the pre- and post-treatment with *z*-score of >1.959, which is the equivalent of a *p*-value of <5%.

## Patient Treatment and Outcome

The patient was evaluated by a pain physician specializing in temporomandibular disorders (TMD), and recorded a pain score of 8/10 at the time of the first scan. The treatment for this subject with severe myofascial pain of the temporomandibular region used botulinum toxin injections, administered at another site 2 days after the first scan. There are extensive studies describing the efficacy and safety of botulinum toxin for TMD (Mor et al., 2015; Munoz Lora et al., 2019). The procedure followed a standard clinical protocol of 10 botulinum units (BU) per injection site. Botulinum was administered to three sites spread 1 cm apart in the masseter and temporalis muscles on each side. The subject reported significant pain relief from the treatment.

The patient's TMJ pain resolved within 24 h of treatment and the pain score was 0/10. The patient was re-scanned 6 days later when the pain score remained at 0/10.

## Results

**Figure 2:**
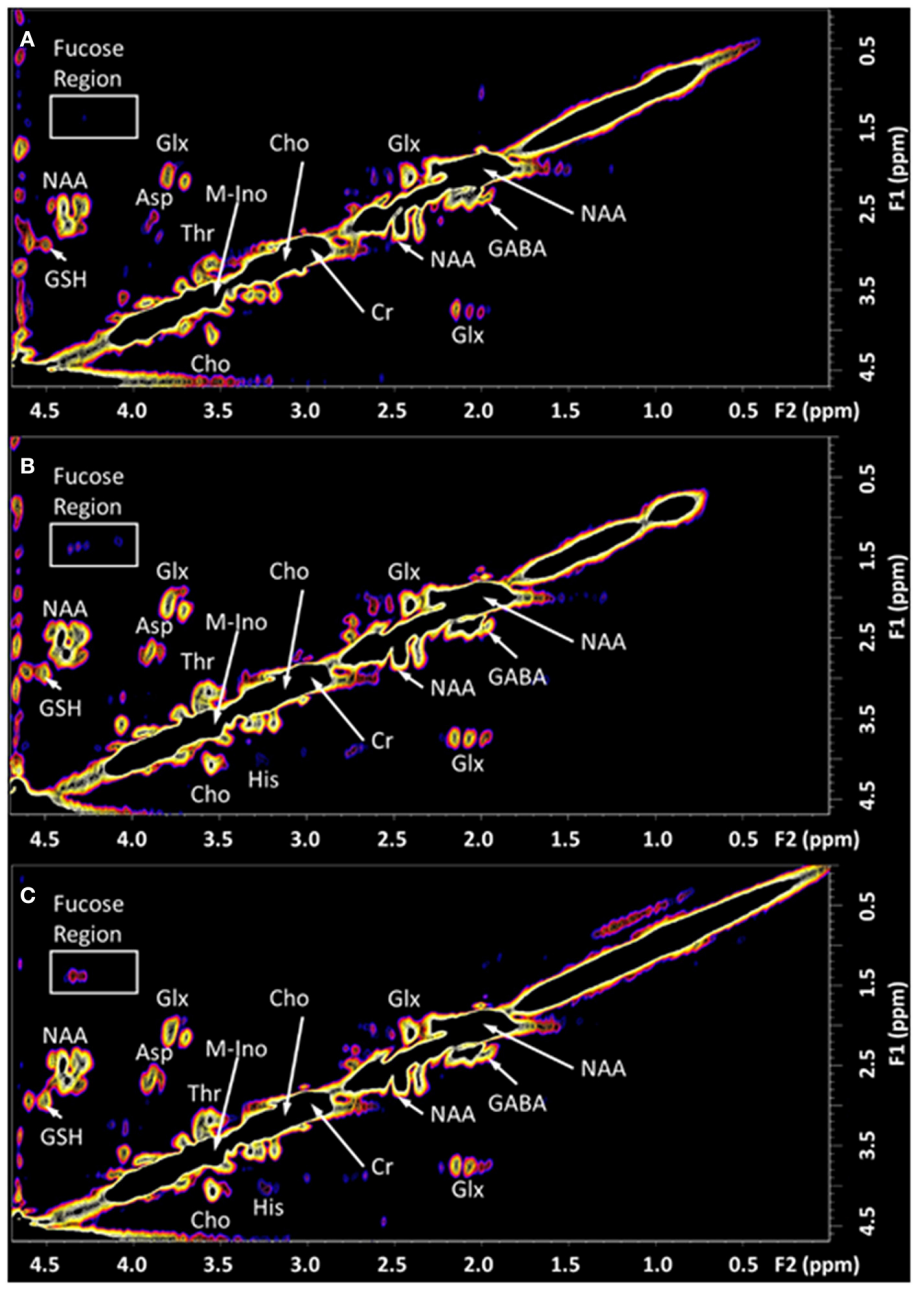
The *in vivo* L-COSY acquired from the PCG region of the brain of a healthy control and the TMJ patient pre and post-treatment. The data was recorded using a 3T clinical scanner (Prisma) equipped with a 64 channel head and neck coil. See methods section for acquisition parameters. **(A)** Healthy control; **(B)** Patient with Chronic Temporomandibular Joint (TMJ) Pain (Score 8/10) before treatment; **(C)** Six days after treatment with Botulinum Toxin A therapy with a pain score of 0/10. NAA, N-acetyl aspartate; Cho, choline; Cr, creatine; Glx, glutamate and glutamine together; Asp, aspartate; m-Ino, myo-inositol; GABA, γ-aminobutyric acid; GSH, glutathione; Thr, threonine. The region highlighted by the white box contains the cross-peaks assigned to the fucosylated glycans and is expanded in Figure 3.

**Figure 3:**
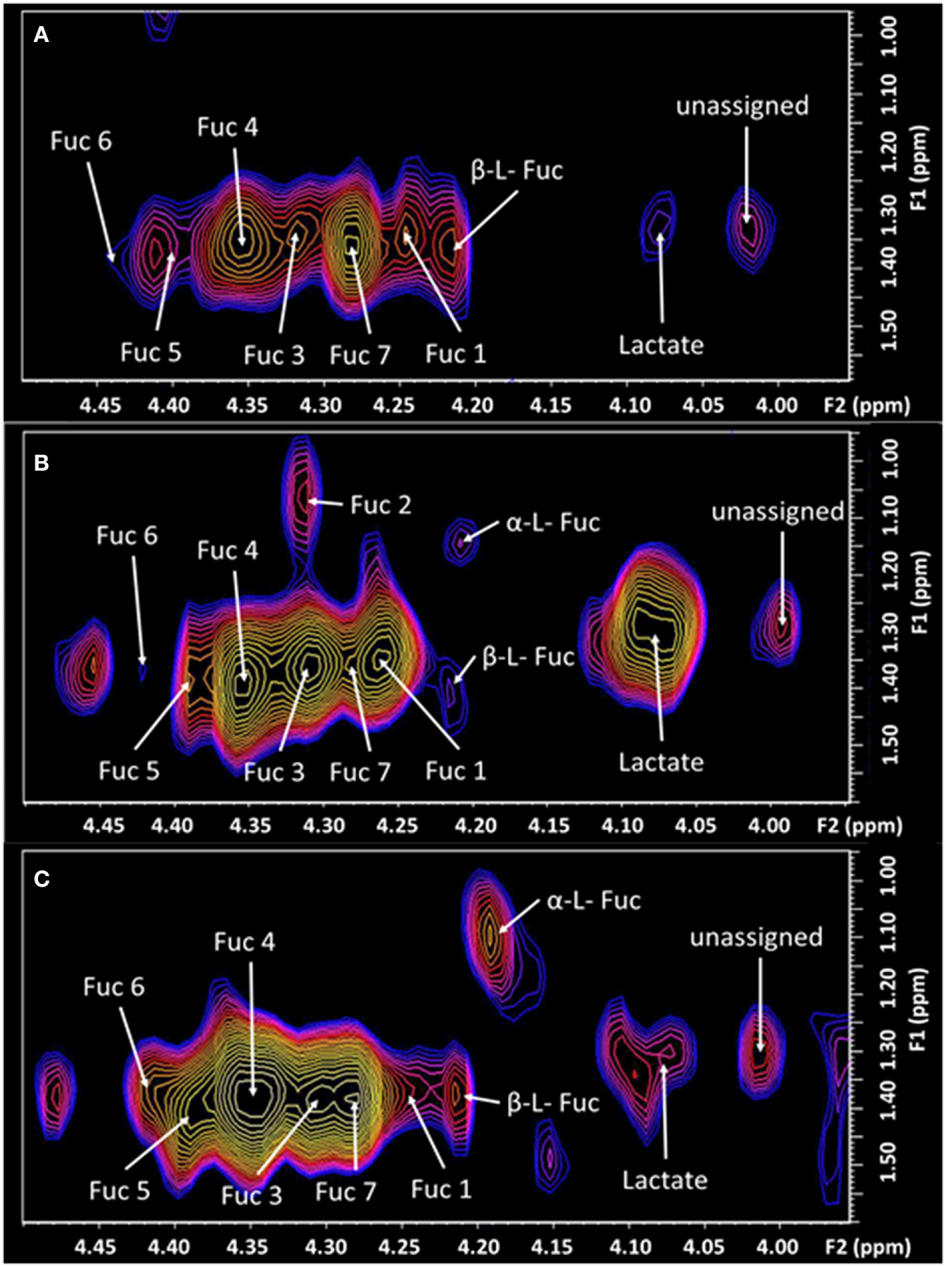
The expanded region (F2: 3.95–4.5 ppm, F1: 0.95–1.6 ppm) of the L-COSY spectrum, shown in Figure 1, with the assignments of Fuc 1 to Fuc 7 denoted. α-L-fucose resonances and lactate are also visible. See Table 1 for chemical shifts. **(A)** Healthy control; **(B)** prior to treatment—pain score of 8/10. Fuc 2 is very intense with pain at 8/10 but absent post-therapy when the pain score was 0/10. Prior to treatment Fuc 6 at 4.44–1.37 ppm is barely visible. **(C)** Six days post-treatment with a pain score of 0/10.

The 2D COSY spectra reporting on the neurochemistry of the patient before and after treatment is shown and compared with typical healthy control spectrum (*n* = 14) in Figures 2, 3. The crosspeak volumes were measured for the 14 healthy pain-free, gender matched controls and the mean compared, with that recorded for the pain patient, before and after treatment in Table 1.

**Table 1:** Neurochemical changes in the TMJ participant pre and post-treatment compared to (*n* = 14) pain free healthy controls, in the posterior cingulate gyrus identified with 2D COSY.

**Neuro-chemical**	**Chemical shift**	**Pre-therapy % diff**	**Pre-therapy**	**Post-therapy % diff**	**Post-therapy**
	**(F2, F1) ppm**	**to healthy controls**	***p*-value**	**to healthy controls**	***p*-value**
Glutamine/Glutamate (Glx)	3.75, 2.10	25.26	0.03^*^	12.50	0.36
Glut Cysteine Moiety	8.13, 8.13	−44.17	0.06	−17.06	0.48
Glutathione	2.52, 2.10	−2.17	0.94	-23.41	0.04^*^
Myo-Inositol	4.05, 3.58	24.29	0.03^*^	−1.86	0.76
Threonine	2.48, 3.62	−15.42	0.40	-31.92	0.04^*^
Lactate	4.08, 1.31	105.10	0.02^*^	41.66	0.44
CH_3_ Lipid	0.91, 0.91	0.76	0.70	204.95	0.002^*^
Unassigned	1.37, 1.99	43.74	0.01^*^	−3.03	0.83
**Fucose region**	
β-L-fucose	4.18, 1.40	−39.01	0.11	5.05	0.82
α-L-fucose	4.21, 1.14	−18.67	0.45	79.69	0.03^*^
Composite Thr/Fuc-α(1–2) glycan denoted “Fuc 1”	4.25, 1.36	43.88	0.06	12.77	0.77
Tentatively assigned to Fuc-α(1–2) precursor or substrate	4.28, 1.13	27.99	0.22	11.23	0.57
Fucose α(1–2)glycan “Fuc 3”	4.31, 1.36	1.08	0.82	16.24	0.15
Fucose α(1–2)glycan “Fuc 4”	4.36, 1.36	16.06	0.76	60.18	0.02^*^
Fucose α(1–2)glycan “Fuc 5”	4.40, 1.37	−21.85	0.32	16.40	0.30
Fucose α(1–2)glycan “Fuc 6”	4.44, 1.37	−46.79	0.09	−23.78	0.48
Fucose α(1–2)glycan “Fuc 7”	4.29, 1.36	27.53	0.26	29.01	0.23

* *The highlighted p-values indicate statistically significant differences (p < 0.05)*.

The TMJ participants' first scan, with a pain score of 8/10 showed a range of differences to the control cohort. The cysteine moiety of glutathione was reduced by 44%. Lactate was significantly increased over 100%. Glx (glutamine and glutamate combination), and the unassigned resonance at (F2, 1.37; F1 1.99 ppm), were significantly increased by 25 and 43%, respectively. The resonance at 1.37 ppm has been consistently reproducible, although there is a risk that it may be attributable to noise from the strong lipid signal at 1.2 ppm. Myo-Inositol was also significantly increased by 24%. The fucosylated glycans, Fuc 5 and Fuc 6 were reduced by 21 and 46%, respectively. The two crosspeaks assigned to the substrate free fucose were reduced by 39 and 18%. The other fucosylated glycans were increased as shown in Table 1.

Six days following the successful treatment, the glutathione cysteine moiety had increased by 27%, but was still 17% below that of the healthy controls. Glutathione (GSH) and Threonine decreased to levels significantly below healthy controls, while Lactate, Glx, myo-Inositol and the unassigned resonance were seen returning to healthy control levels. The CH_3_ lipid increased significantly above healthy control levels. The α-L-fucose substrate 1, had almost reached equilibrium but α-L-fucose substrate 2 had increased from 18% below healthy controls to 80% above healthy controls albeit with a *p*-value of only 0.82. Fuc 4 increased significantly to 60% above the level of healthy controls. Fucosylated glycans Fuc 1 and 2 had almost returned to healthy control levels, 6 days after treatment. Fucosylated glycan Fuc 7 remained elevated while Fuc 3 and 6 increased. However, Fucosylated glycan Fuc 6 was still 23% below healthy controls. Fucosylated glycans Fuc 5 had increased from 21% below healthy controls to 16% above the healthy controls.

## Discussion

This is the first report of a detailed neurochemical response of the human brain, following successful treatment for pain. It provides an insight into the neurochemical pathways affected by the high level of pain and how the brain is attempting to readjust after the pain was treated. It is commonly reported that when a person has chronic pain, they have difficulties with cognitive functioning and are unable to concentrate (Jamison et al., 1988; McCracken and Iverson, 2001; Dick and Rashiq, 2007). The neurochemical information recorded in this patient, provides some insight into these reports and is consistent with the report that the fucosylated glycans are involved in neuronal function (Ralf and Melitta, 2004; Murrey et al., 2009). The 2D COSY method recorded the response of the human brain to therapy and indicates that the kinetics of the repopulation of these Fuc-α(1–2) glycans can also be monitored.

Whilst the CH_3_ lipid was found to be significantly increased when compared to the healthy controls this may have been due to voxel placement. If the voxel is incorrectly placed too closely to the skull, lipid contamination may arise from the skull, subcutaneous tissues and meninges (Mountford et al., 2010).

Level of Fuc-α(1–2) glycan denoted Fuc 2 is considered a marker of pain intensity (Manuscript under review). Thus, recording a return to normal of this glycan, when the pain level reduced from 8/10 to 0/10, is consistent with this finding. Glycans have important recognition roles in neuronal regulation and are important in the metabolic requirements of the peripheral and central systems (Hayes and Melrose, 2018).

Whilst the patient was in pain the substrates α-L-fucose were severely depleted in the brain, compared to the control cohort. After treatment, high levels of α-L-fucose were recorded in this patient, which appears to be the brain attempting to repopulate the affected glycans. Fucosylated glycoproteins control transmission of synaptic neurotransmitters and neural function (Hayes and Melrose, 2018).

Carbohydrates are able to encipher distinct information, which is recognized by receptors and translated into specific biological processes, due to the inherent variance of the glycan structures. Within the brain, it is considered that fucose is found within the synapsin proteins (Hart, 2006), which are involved in the regulation of releasing neurotransmitters at the synapse (Evergren et al., 2007), with the fucosylation preventing the expeditious reduction of these proteins (Hart, 2006). It has been established that blocking the fucosylation of synapsin Ia/Ib, has the ability to affect the hippocampus, by suppressing the glucocorticoid-mediated increase in stress-related memories (Revest et al., 2010).

There are currently seven Fuc-α(1–2) glycans currently assigned (Table 1) and able to be monitored in a clinical 3T MR scanner, with a 64 channel head and neck coil. It is likely that further substrates will be identified and will be linked to specific glycans repopulating. There remains much to be undertaken to comprehensively map brain neurochemistry with this novel method.

**Figure 4:**
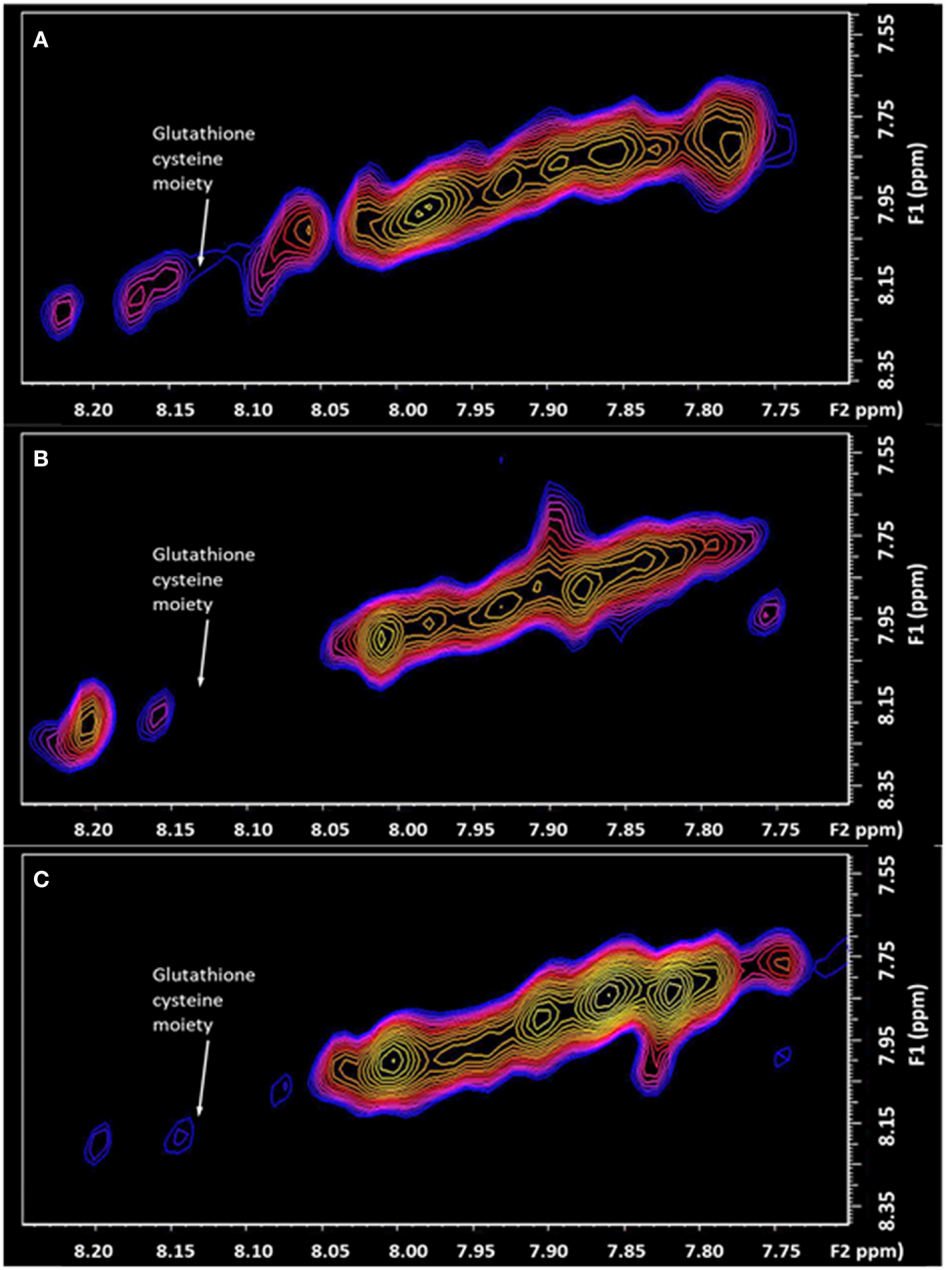
The expanded region (F2: 7.70–8.25 ppm, F1: 7.50–8.40 ppm) of the L-COSY high frequency region, with the assignment of the Glutathione cysteine moiety denoted (F2: 8.13, F1: 8.13 ppm). **(A)** healthy control; **(B)** Patient with Chronic Temporomandibular Joint (TMJ) Pain (Score 8/10) before treatment; **(C)** Six days after treatment with Botulinum Toxin A therapy with a pain score of 0/10.

Important is the large depletion of the diagonal resonance at 8.13 ppm of 44% in the chronic pain state (Figure 4). This diagonal resonance has thus far been assigned to the glutathione cysteine moiety. If this assignment is correct, it is indicative of a glutathione redox imbalance, as a consequence of the chronic pain. Such an imbalance has been suggested in animal models, to be an early marker of neurodegeneration (Aoyama and Nakaki, 2013). This molecule is seen to be returning halfway back to the healthy control level, 6 days after the successful treatment by Botulinum Toxin A. The progressive decline of neuronal function in the central or peripheral nervous system, and the eventual death of nerve cells, is representative of neurodegenerative diseases. This links directly to the very large changes recorded in the Fuc-α(1–2) glycans, known to be located at the end of the neuron (Hsieh-Wilson, 2001).

The capacity to monitor the effect of pain on the human brain and how it responds to successful treatment, is the first step in a personalized approach to monitoring therapy objectively. It also provides strong clues as to the biochemical pathways affected by chronic pain, and how they recover. An objective measurement of pain could help in discriminating the relative contributions in the complex interplay of the sensory and affective (emotional suffering) components of pain, leading to appropriate individualized pharmaceutical drug regimens.

## Data Availability Statement

The data that support the findings of this study are available on request from the corresponding author CM. The data is not publicly available due to privacy and consent concerns raised by ethical boards.

## Ethics Statement

The studies involving human participants were reviewed and approved by Queensland Health Metro South Ethics Committee (HREC/17/QPAH/808). The patients/participants provided their written informed consent to participate in this study. Written informed consent was obtained from the individual(s) for the publication of any potentially identifiable images or data included in this article.

## Author Contributions

CM, GG, and JW: study conception and design. JW and EV: acquisition of data. CM, DL, GG, and JW: analysis and interpretation of data. JW and CM: drafting of the manuscript. CM, DL, EV, GG, and JW: critical revision. All authors contributed to the article and approved the submitted version.

## Conflict of Interest

CM has filed patent registrations in this area. The remaining authors declare that the research was conducted in the absence of any commercial or financial relationships that could be construed as a potential conflict of interest.

## Publisher's Note

All claims expressed in this article are solely those of the authors and do not necessarily represent those of their affiliated organizations, or those of the publisher, the editors and the reviewers. Any product that may be evaluated in this article, or claim that may be made by its manufacturer, is not guaranteed or endorsed by the publisher.
